# Editorial: Risk and protective factors, family environment and (a)typical neurodevelopmental outcomes, volume II

**DOI:** 10.3389/fpsyg.2026.1771469

**Published:** 2026-04-08

**Authors:** Valentina Riva, Giorgia Bussu, Anna Gui

**Affiliations:** 1 Scientific Institute, IRCCS E. Medea, Child Psychopathology Unit, Lecco, Italy; 2 Sence Research AB, Uppsala, Sweden; 3 Department of Systems Medicine, University of Rome Tor Vergata, Rome, Italy; 4 Centre for Brain and Cognitive Development, Birkbeck University of London, London, United Kingdom

**Keywords:** developmental trajectories, family and caregiving environment, neurodevelopment, neurodivergent population, risk and protective factors

Child development emerges from a non-linear interplay of biological predispositions, early skills, and environmental contexts. Developmental neuroscience and neuroconstructivism emphasize that neurodevelopmental phenotypes reflect how the maturing brain adapts to early experiences (Karmiloff-Smith, 2006; Johnson, 2017). As children's primary environment, families play a central role through sensitive and responsive caregiving (Wilder and Semendeferi, 2022; Halkola et al., 2025). Crucially, recent evidence suggests that family resilience, as in the capacity to maintain hope and collaborative problem-solving, acts as a primary protective factor, often outweighing individual clinical markers in predicting long-term success (Cameranesi et al., 2022).

Volume I of this Research Topic (Provenzi et al., 2023) introduced this ecological perspective on early development, highlighting the role of caregiving environments, parental wellbeing, and early relational experiences in shaping neurodevelopmental trajectories.

In continuity with Volume I (Provenzi et al., 2023), Volume II extends this ecological perspective by integrating evidence across diverse populations and contexts, capturing the heterogeneity of early development and the interplay between risk and protective factors (Riboldi et al., 2024).

This Research Topic includes 10 articles: seven empirical studies, one theoretical synthesis, one mini-review, and one systematic review. The empirical contributions adopt longitudinal, cross-sectional, multi-informant, and qualitative designs and draw on samples from multiple geographic and sociocultural contexts, including Southern and Northern Europe and East Asia, to examine early development, parental wellbeing, family dynamics, in neurotypical and neurodivergent populations.

## 1 Early mechanisms of learning, language, and communication

A first group of articles focuses on early mechanisms of learning, language and communication in social contexts. Ishikawa and Itakura demonstrate the crucial role of early environments in the development of social learning. This process relies on ostensive cues in infancy, shifts toward learning driven by the intrinsic reward value of social actions in toddlerhood, and culminates with selective learning, where older children learn from competent, conventional, and in-group models.

Language development is examined in Lozano et al., who adopt a female-centered perspective, proposing that early selective attention to the articulating mouth may function as a sex-specific protective mechanism, contributing to more favorable language trajectories in females at elevated likelihood for autism. Complementing these theoretical approaches, Capelli et al. provide evidence linking early attention and language learning in an Italian cohort. They show that greater auditory distractibility during parent–child interaction at 12 months predicts poorer language outcomes at 24 months, and provide preliminary evidence for a protective role of joint attention for vocabulary development.

Taking a different perspective, Palomero-Sierra et al. explore early socio-communication difficulties as a risk factor for cognitive and language development in Spanish moderate-to-late preterm children. They showed that pointing, responding to joint attention, and attending to sounds at 1 year of age predict receptive and expressive language at 2 years, confirming the importance of early socio-communication screening in this population.

Jurišová et al. examine developmental trajectories in Slovakian infants born at extremely low gestational age, reporting a clear decline in cognitive, language, and motor functioning across the first 2 years, with language showing the greatest vulnerability. Although some infants maintained stable development, others presented persistent or emerging delays, particularly those born at the lowest gestational ages and birth weights.

## 2 Family processes, wellbeing, and caregiving environments

Family processes and caregiving environments represent a second major theme of this Volume. Using the coparenting ecological model, Wang et al. show that higher co-parenting quality is linked to better socio-emotional competencies in Chinese young children, partly mediated by parental stimulation. Their findings underscore coordinated parenting as a protective factor and highlight how family wellbeing and psychological states shape perceptions of neurodevelopmental phenotypes. Perzolli et al. provide evidence of specific mental health patterns and perceptual divergence in Italian mothers and fathers of autistic children. Their findings indicate that while parental stress levels are comparable across parents, mothers report higher clinical mental health symptoms, such as anxiety and depression. Crucially, these psychological states seemed to function as a “perceptual lens”: maternal caregivers reported more severe internalizing and externalizing behaviors in their children compared to paternal reports.

Complementing this, Wong et al. extended the focus beyond the parent-child dyad to the broader family ecosystem in the context of Parent-Implemented Interventions in Hong Kong. Their qualitative findings show that extended family involvement ranges from rejection to full embracement of intervention strategies. High levels of familial “embracement” may function as a strong protective factor, creating a consistent nurturing environment that bolsters the child's social communication skills.

## 3 Sociocultural contexts and broader caregiving systems

Further contributing to the study of non-Western contexts, Jia-Yuan et al. interviewed Chinese children and their primary caregivers to identify which aspects of family care undermine children's basic developmental needs, such as daily living care, emotional and psychological support, safety and educational guidance. Their qualitative analysis revealed that family-level factors and broader social pressures jointly constitute risk factors for children's outcomes.

Finally, Battistin et al. synthesize the limited yet important literature on sibling relationships in children with vision impairment. Despite warm and supportive bonds, siblings may face emotional challenges, reflecting how disability reshapes family dynamics.

## 4 Conclusions

Volume II extends the multidisciplinary perspective of Volume I, demonstrating that early neurodevelopment emerges from the interaction between child characteristics, family processes, and sociocultural contexts. Further progress requires cross-cultural and integrative designs combining biological, behavioral, and contextual data to clarify how developmental trajectories unfold over time. By considering fathers, siblings, and extended family members, this volume highlights the importance of a broader family perspective, aligning with multisystemic resilience frameworks (Cameranesi et al., 2022). As neurodevelopment is increasingly understood as a transactional process, future research should account for how parental “state-dependent” reports and household alignment interact with biological factors. From a public-health perspective, these findings emphasize the value of family-centered interventions that support the entire household. Integrating paternal mental health screening and extended family involvement can help create more holistic, culturally sensitive services that promote resilience (Le Bas et al., 2025). Inspired by the promising findings of the longitudinal articles in this Research Topic, it will be important to invest resources in longer follow-up periods to elucidate developmental trajectories from infancy into later childhood. These efforts will benefit from multidisciplinary and culturally sensitive approaches and from theoretical frameworks that connect multiple levels to advance the field.
